# Health outcomes of the July 14, 2016 Nice terror attack among hospital-based professionals and students: the « ECHOS de Nice » health survey protocol

**DOI:** 10.1186/s12889-019-7489-3

**Published:** 2019-08-23

**Authors:** Laurence Bentz, Philippe Pirard, Yvon Motreff, Stéphanie Vandentorren, Thierry Baubet, Roxane Fabre, Pia Touboul Lundgren, Christian Pradier

**Affiliations:** 1 Public Health Department, Cote d’Azur University, Nice University Hospital, F-06200 Nice, France; 2 Non-communicable Diseases and Trauma Division, French National Public Health Agency (Santé Publique France), F-94415 Saint-Maurice, France; 3 Epidemiology and Population Health Research Centre (CESP), National Health and Medical Research Institute (INSERM), Unit 1178 “Santé Mentale et Santé Publique”, F-94807 Villejuif Cedex, France; 4 Department of Social Epidemiology, National Health and Medical Research Institute (INSERM), Pierre Louis Institute of Epidemiology and Public Health (IPLESP), Sorbonne University,, F75012 Paris, France; 5 French National Public Health Agency (Santé Publique France), Regional Office of Nouvelle Aquitaine, F-94415 Saint-Maurice, France; 6 Paris-Seine-Saint-Denis University Hospitals (AP-HP), Hôpital Avicenne, Paris 13 University, F-93000 Bobigny, France; 7 National Resources and Resilience Centre (CNRR), F-93000 Bobigny, France; 8 CoBTeK lab, Nice University Hospital, CMRR, Cote d’Azur University, F-06200 Nice, France

**Keywords:** Terrorist attack, Hospital staff, Stress disorders, Post-traumatic, Needs assessment, Research design, Occupational health, Web questionnaire

## Abstract

**Background:**

The terror attack of July 14, 2016, in Nice, France, resulted in 86 deaths, including children, and several hundred wounded, with a major psychological impact on the population. Hospital staff had to cope with exceptional circumstances which made them vulnerable to detrimental effects on their own health.

This paper describes the method that was selected for the survey entitled “*ECHOS de Nice 14 Juillet*” which aimed to assess the impact of the attack on the psychological, psycho-traumatic and somatic health condition of the Nice University and Lenval hospital staff who were directly or indirectly exposed to the attack, and also to describe the support and care facilities they were offered.

**Method:**

*ECHOS de Nice 14 juillet* is an observational, cross-sectional, multicentre study focusing on all the hospital staff and students of both institutions, i.e. 10,100 persons in June 2017. A web-based questionnaire based on the model developed by Santé Publique France (*IMPACTS* and *ESPA 13 novembre 2015*) was adapted to the contexts of the healthcare professionals and students employed in these healthcare institutions in Nice and published on line from June 21 to October 30, 2017. The paper describes the tools that were used to meet the aims of the study, i.e. identification of exposure categories (‘civilian’ exposure for those present during the attack and/or ‘professional’ exposure); indicators of psychological impact (anxiety, depression, burnout, compassion fatigue, suicidal states, tobacco and alcohol use, self-medications), psycho-traumatic and somatic impact; professional and social impact. Lastly, awareness of availability and use of psychological support and care-follow-up facilities by professionals were investigated. Respondents could include extensive qualitative comments on the various themes explored in the questionnaire, with text analysis complementing that of quantitative data.

**Discussion:**

The benefits and limitations of the selected methodology are discussed, in view of contributing useful information to help anticipate and manage health issues among hospital staff who have been victims of traumatic events.

## Background

The July 14, 2016 terror attack in Nice, during the Bastille day fireworks, when a lorry rammed into the crowd, resulted in many victims among the civilian population: 86 dead, among whom 10 children and adolescents, and 434 wounded [1]. Thousands were involved in the event. A great number of healthcare professionals from all staff categories were also directly affected by the attack: either on the site of the attack as civilians attending the fireworks, or as hospital healthcare professionals taking charge of the victims and/or their families. Two healthcare institutions were involved in the immediate aftermath of the event and the long-term follow-up of victims: the Lenval paediatric hospital which was the first institution to admit both children and adult victims, and the Nice University hospital. In such a context, healthcare professionals were faced with particularly challenging circumstances, with a major influx of victims both living and deceased, including children, severe injuries [2], exceptional clinical and psychological management procedures for victims and their families, and the search for those missing [3].

This type of event places healthcare professionals from the institutions at risk of developing psychological and/or somatic disorders related to the event. Compared to natural or technological causes, intentional mass violence has been shown to have an even greater impact on populations in terms of psychological distress [4]. Repercussions may be felt in many areas of daily life, affecting professional, family and social functioning [5].

To our knowledge, few studies have focused specifically on health outcomes among hospital staff and their care and support-seeking practices in the aftermath of the event.

Regarding the psychological component, this type of event can induce depression-related anxiety disorders [6–10], panic attacks [11], and suicidal thoughts, behaviours and/or fatalities among care-providers [12–14].

Caregivers may also develop post-traumatic stress disorder (PTSD) which is defined in adults by a range of criteria [15]. The first of these is exposure to a traumatic event that occurred outside the scope of usual human experience [16, 17]. Diagnostic criteria for PTSD include those related to the exposure (criteria A1 to A4) and those related to the presence of four symptom categories (criteria B to E) associated with the traumatic event and arising within the first month following the event. Patients may also display dissociative features (depersonalisation, derealisation). In PTSD, these disorders last for more than a month [15, 18, 19], and may progress to chronic PTSD when symptoms persist beyond 3 months [15]. Onset of PTSD may be delayed, i.e. full diagnostic criteria are not met until at least 6 months after the trauma(s), although onset of symptoms may occur immediately [15]. These disturbances cause clinically significant distress or impairment in social, occupational or family or other important areas of functioning and are not attributable to other causes than trauma exposure [15, 18, 19].

Such consequences vary according to participants’ occupation. Strong variations in prevalence of PTSD are described in the literature according to the type of occupational and exposure situations. [20]. In the immediate aftermath of attacks or mass shootings, on-site rescue workers are the first to intervene: prevalence rates among these rescuers ranges from 0.4 to 22.0% [21–25]. Among rescuers who were health professionals involved in the November 2015 terrorist attack in Paris, the observed prevalence rate was 4.5% [10]. These studies thus confirm that first responders and/or rescue or recovery workers are at high risk for PTSD [26], but prevalence rates are not sufficiently documented for health professionals who intervene at a later stage of the care process, and more broadly, for hospital staff.

Furthermore, specific types of professional traumatic events and associated levels of exposure are still not established [27]. In the context of the Nice terror attack, exposure among professionals from Nice University and Lenval hospitals included confronting an exceptionally stressful environment, with a major influx of victims both living and deceased and a number of tasks widely exceeding their usual work situations [28]. Health professionals were exposed either directly (primary trauma, through their presence on the site of the attack; or according to the type of management procedures that may be considered as trauma) or indirectly, through secondary exposure to individuals (victims, family members or colleagues) who had witnessed highly stressful or traumatising events (vicarious trauma). Trauma related to hospital work can be considered a combination of primary and secondary trauma [29].

Although full PTSD-defining B-E criteria may not be systematically present in trauma victims, symptoms can be a source of distress. This has been characterised as sub-threshold PTSD [30]. The prevalence rate thus ranges from 8 to 24% according to the population under study [24, 31–34]. As these sub-threshold conditions may be clinically significant and result in incapacitating social, professional and functional impairment [35], they should therefore be identified and taken into account so that therapeutic assistance can be offered to those with these conditions.

Healthcare professionals are at risk for compassion fatigue. This exclusively concerns professionals involved in various forms of assistance (first aid professionals, clinicians, social workers …) [36]. The term refers to symptoms which appear to result from exposure to the difficulties confronted by patients and the permanent burden of the empathy they require. Thus, the compassion fatigue that healthcare professionals may experience includes burnout and secondary traumatic stress (vicarious trauma) [29]. Secondary traumatic stress results from secondary exposure of professionals to individuals who have lived through highly stressful or traumatising events. In such circumstances, it can lead to sleep disturbance, intrusive images, avoiding reminders of the patient’s traumatic experience. Although most studies on this syndrome have focused on clinicians treating psychological trauma among survivors [37], this syndrome has been identified for several years among therapists working with victims and their families [36, 37].

Increased use of tobacco, alcohol and marijuana may be related to the psychological outcomes linked to a terrorist attack, sometimes over a prolonged period [38]. These types of substance use were also found to be associated with depression or PTSD [39]. They have been identified among a significant proportion of agents who had worked on the World Trade Centre site [40] or among Norwegian rescuers who intervened in 2011 [41]. Such increased use was confirmed in the first 2 years following a terrorist event [42].

Besides the consequences at the professional level, a disaster may also have a negative somatic impact [6, 7]. Following terrorist attacks, somatic disorders were observed among rescuers in the form of heart disease [43, 44], arising in various contexts [40, 41]. More specifically, patients presenting with PTSD have a significantly higher risk of developing diabetes [45], sleep disturbances [46], cardiac and auto-immune disorders [7, 47, 48].

Repercussions on care-givers can be experienced in all aspects of life, including in the social and family environment [49]. Physicians in London suffering from multiple co-morbidities (depression, alcohol abuse, anxiety and self-medication) described greater difficulties in adapting to daily life, with repercussions on their professional activity [5].

However, when care programmes are implemented, follow-up has a positive impact on both social and professional capacities, making it possible to resume work [50]. In the *IMPACTS* study, just over half the protagonists received medical and psychological care in the aftermath of the attacks, mostly within their institution. [9]. It is thus worth investigating, as a complement to exploring psychological and somatic repercussions, the awareness and use of hospital support facilities that are provided in the aftermath of a terror attack.

The objective of this paper is to describe the method that was selected for the *ECHOS de Nice 14 juillet* study which aimed to estimate the impact of the terror attack on the psychological, psycho-traumatic and somatic health of hospital staff and students at Nice University and Lenval hospitals who were directly or indirectly exposed to the July 14th attack, and to describe hospital professionals’ use and needs regarding support and care facilities.

## Methods/design

### Study design

This was a cross-sectional, multicentre, observational study.

### Study population

The study focused on all hospital staff and students above 18 years of age registered with Nice university and Lenval hospitals from July 13, 2016, whether directly or indirectly involved in the terror attack of July 14, 2016. Overall, 10,100 subjects were registered on the Human Resources Departments’ lists of both hospitals in June 2017.

### Non-inclusion criteria

Those below 18 years of age when completing the questionnaire, as well as those not or no longer employed as hospital staff, medical students and residents, or paramedical students by the institutions concerned between July 2016 and the beginning of the study.

### Study period

Participants were asked to complete a secured web-questionnaire available online from June 21 to October 30, 2017.

### Ethical considerations – informed consent – IT security

The study was registered with the French Data Protection Authority under N°270 and with the Ethics Committee (CPP Nord ouest III, Caen, France) under N° ID RCB: 2017-A00812–51. Specific measures were implemented to guarantee IT security of collected data. The implementation process of the *ECHOS de Nice* web questionnaire was identical to that of a study focusing on victims of the November 2015 terror attack *(ESPA 13 novembre*) [10, 51] which had been subjected to an external security audit.

Information regarding the objectives and the procedures related to the survey were available on the intranet sites of both institutions. A letter was attached to the payslip of each member of staff in June 2017. Specific communication activities were conducted among the teams of various departments and staff representatives.

Access to the website also facilitated access to information on psychological trauma and its determinants. [http://invs.santepubliquefrance.fr/Dossiers-thematiques/Populations-et-sante/Actes-terroristes].

Lastly, the questionnaire gave details of psychological support facilities available within the institutions (helpline, secretarial office hours for making appointments). Medical students were informed that they could request support via an email address. All respondents who wished to provide contact details in order to be contacted by a psychologist specialised in psychological trauma could do so, and a hotline was also established to enable them to call the specialist directly.

Lastly, inclusion data and survey data were stored in separate databases.

### Web-based questionnaire

The questionnaire was specifically adapted for health professionals and students employed in health institutions in the Nice context. To take health-related literacy into account, particular care was given to the wording of the questions to facilitate their comprehension [52]. The questionnaire was pilot-tested on the various categories of professionals from several hospital departments, with varying degrees of exposure to the attack, to record their comments and suggestions for change. Filters were developed in such a way that questions were adapted to the respondent’s situation (hospital staff or student, involved or not in the attack and its aftermath). Completing the questionnaire was estimated to require approximately 20 to 45 min.

### Collected data

#### Socio-demographic and professional information

The psychological and somatic disorders resulting from the terror attack among involved members of staff were analysed according to age and gender, institution and department of activity at the time of the attack (Nice university and Lenval hospitals), years of professional experience, professional category (as described by the Human Resources department, i.e. medical, caregiver, administration, logistics/technical, medico-technical, socio-educational staff).

The impact may indeed differ according to the professional category of exposed professionals, (e.g. nurses, doctors, [53], psychologists [54], ambulance staff) [55, 56]. It may also vary according to the hospital department with potentially severe psychological repercussions among rescue workers or emergency department staff who are regularly faced with traumatic events in their professional environment. [21, 56–58]. These repercussions may also result from an exceptional exposure of members of staff usually less liable to be confronted with such situations and less trained in the management of psycho-social risks (e.g. ambulance staff).

These socio-demographic characteristics contribute to the analysis of known risk or protective health-related factors. Namely, it will thus be possible to compare levels of PTSD with published data according to professional categories and to measure their correlation with the level of exposure and other determinants.

#### Circumstances and degree of trauma exposure

Collected data included site of exposure (on-site or at a later stage), sensory exposure (according to the definition of trauma which always results from a perception or sensation, i.e. having seen, heard, smelt), [59], the timing of professional intervention (early intervention on the attack site for rescue staff, and/or intervention within the hospital departments) [60], the nature of professional tasks performed in relation to the circumstances of the attack. A description of acute stress [61] and/or peritraumatic experience at some point during exposure [62] were considered risk factors for PTSD [63].

#### Professional tasks performed by hospital staff

Exposed professionals were questioned on the circumstances of their professional intervention. Professional tasks were grouped into three major categories:

- Tasks related to living or dead bodies (clinical, surgical, forensic, but also transportation of living or deceased victims, X-rays of bodies or body parts, cleaning, forensic activities …).
- Tasks related to the psychological management of victims and families, informing families of deceased victims, communicating and supporting persons in distress (comforting, hosting, translating for foreign families); support for distressed professionals and students.
- Tasks related to crisis management (triggering the emergency plan, …), processing of phone calls for emergency services, management of operational hospital back-up (technical, logistical), identification and follow-up of victims during the course of their care pathway, administrative or logistic details (update and follow-up of records for those in care, etc. …).

Other questions focused on familiarity with the professional procedures undertaken and/or whether these tasks were part of usual duties; prior training before they were performed; circumstances giving rise to special difficulties when performing professional tasks. All these items were liable to compound the impact of professional exposure.

#### Staff exposure categories

Staff exposure was categorized according to criterion A which contributes to the definition of PTSD in adults [15]. Criterion A identifies the trigger to PTSD as exposure of an individual to threatened death, severe injury or « first-hand repeated or extreme exposure to aversive details of the traumatic event ». This last situation may concern first-line rescuers gathering human remains. Aside from such specific circumstances, this exposure category may have applied to a wide range of situations and activities, including staff directly involved with victims, but also any professional category subjected to intense operational pressure.

According to circumstances of exposure, three groups were identified:

- Those members of staff exclusively involved: these were not present on the site of the attack but were involved during a specific stage of victim assistance or of institutional organisation in relation to assisting victims.
- Those having witnessed the attack, regardless of whether they were involved or not in assisting the victims.
- Those not exposed, nor having witnessed the attack and not involved in any stage of assistance to victims.

#### Psychological impact indicators

##### Anxiety and/or depression

Hamilton developed a double rating scale (Hospital Anxiety and Depression scale, HAD) measuring the intensity of perception of seven symptoms indicative of anxiety (7 questions scoring 0 to 3) and of seven symptoms indicative of depression (7 questions scoring 0 to 3) during the previous week. The HAD scale, which has been approved for the general population, provides a screening tool for states of anxiety or depression (confirmed for a score above 9) that calls for specialist assessment [64–67].

##### Suicidal thoughts

Persons exposed to the attack were asked whether the distress experienced since the attack had led to suicidal thoughts; if so, their delay of emergence; how these thoughts developed (fleeting ideation; frequent ideation without an intent; suicidal intent; programming a suicidal gesture; interrupted suicide attempt; suicide attempt); consultations because of these ideas; personal history of suicidal thoughts during the year before the attack, with or without hospitalization. Respondent’s resort to medical help in relation with this issue was also investigated.

##### Compassion fatigue

This breaks into two parts: [29].

The first part concerns reactions such as exhaustion, frustration, anger and depression typical of burnout or professional exhaustion [68].

The second part is a negative feeling driven by fear and work-related trauma, known as « secondary traumatic stress » or « vicarious trauma ». Burnout and secondary traumatic stress were detected and assessed using the ProQOL measurement tool which was developed to identify these issues among helpers [29]. A score of 57 and above indicates the significant presence of each of these symptoms and allows detection of probable anxiety or depression syndromes, respectively. The score is used as a basis for discussion during the clinical examination with the physician. Burnout, secondary traumatic stress, depression and post-traumatic stress syndrome are often combined [29].

Changes in tobacco, alcohol and marijuana use were also investigated among respondents, before and after the terror attack. Respondents were asked whether, since the attack, they had started consuming these substances; had increased, decreased or discontinued their consumption; had a stable consumption; had never started or had discontinued for several years.

Changes in medication following the attack were also explored (medication for sleep disturbances, fear or stress, depression), prior use being considered as a potential pre-traumatic risk factor.

#### Indicators of psycho-traumatic impact

The presence of PTSD was explored on the basis of answers to the PCL5 (PTSD CheckList), which is a scale developed to assess the presence and intensity (from 0 to 4) of the 20 symptoms divided into four categories (criteria B to E) associated with the traumatic event and arising in its aftermath [15, 18, 69–72]:

- Intrusive symptoms (criterion B),
- Avoidance of trauma-related stimuli after the trauma (criterion C),
- Negative alterations of cognition and mood (criterion D),
- Trauma-related arousal and reactivity alterations (criterion E).

The presence of probable PTSD was based on the analysis of symptom categories, of over 1 month’s duration. (criterion F) [30]. For each item, a score of 2 or above is considered clinically significant*,* considering that a provisional diagnosis of PTSD can be made by treating each item rated 2 or higher as an approved symptom and then following the DSM-5 diagnostic rule which requires at a minimum: 1 element of criterion B, 1 element of criterion C, 2 elements of criterion D and 2 elements of criterion E [69].

Respondents were also asked whether symptoms were associated with a stated handicap affecting daily functioning (in their professional, social, family environment) (criterion G) [15].

##### Sub-threshold PTSD

Based on the most common definitions encountered in the literature, McLaughlin suggests defining this condition as applicable to respondents meeting two or three (but not all four) of DSM-5 Criteria B, C, D, or E as described above [30].

### The questionnaire also explored risk and protective factors for PTSD both from the personal and professional perspectives

Pre-trauma factors to be considered included female gender [15], previous trauma exposure [16], prior use of psychoactive drugs [73], type of health-related profession which determine the type of tasks performed and may constitute risk factors for PTSD (emergency workers, paramedics, ambulance staff, etc … ). Assessment of previous trauma exposure was declarative: respondents were asked whether they had previously been confronted with potentially life-threatening events, during which they felt brutally threatened, or their life endangered (serious accident, physical aggression, military combat or war, sexual contact in childhood with an older person, natural disaster, sexual assault or rape, etc.). It also conversely included an investigation of protective factors (physicians being sometimes considered more resilient than other professional categories) [23, 74], prior professional training in view of confronting a critical situation [8], particularly in A&E departments where such situations are regularly encountered [75, 76]. The *ECHOS de Nice* questionnaire thus included questions regarding awareness of psychosocial risks, identification of resource persons, awareness of psychological consequences after involvement in a traumatic event.

To investigate peri-traumatic factors, the level of exposure to the traumatic event was taken into account, with a higher degree of severity for professionals who were directly involved [74], the temporal and environmental circumstances of exposure: first responders, on-site rescuers [9, 24, 77–79] or secondary intervention within an institution [80], the level of environmental safety and the perceived safety level [53, 81], the development of acute stress disorder following exposure to the event [15], for which the Bracha scale provides a checklist by measuring the level of physical symptoms linked to adrenal activation (4 questions scoring 0 to 5) [61]. Lastly, peritraumatic dissociation and its subsequent persistence in the aftermath were explored. The peritraumatic dissociative experience scale was used [62], which measures dissociative experiences during the traumatic event and the following hours with 14 questions scoring from 1 to 5. Those who experience a high degree of dissociation have a higher risk of developing PTSD, presumably because this reflects a defence mechanism when resilience capacity is exceeded in a context of highly intense exposure to a traumatic event. A score of 15 or above is considered to reveal significant dissociation [82].

Among post-traumatic factors, the perceived quality of social support in the aftermath of the traumatic event is a major protective factor [9, 10, 82–84].

#### Somatic impact indicators

On a declarative basis, 13 somatic symptoms were explored in our study. For each symptom, respondents were asked whether they had suffered pain since the attack; if the symptoms were already present before the attack and if they had worsened since; if they were still affected at the time of the study.

#### Indicators of psychosocial impact

##### Professional impact

Exposed health professionals were asked whether they were relieved from their post after their intervention and how they considered this decision, whether their state of health precluded further work or training, and what occupational accident notification or work stoppage prescriptions had been issued. Indeed, 6 months after the Paris November 2015 terror attack, 6% of on-site rescuers stated they were unable to return to work [9]. Moreover, teams confronted with this type of situation were those with the highest turnover, as a proportion of their members chose to move to another department or institution [11].

##### Consequences on social life

Particularly repercussions on families and especially children [3]. We used the Sheehan disability scale, which was developed to assess functional impairment in three inter-related domains: work/studies; social and family life. The respondent rates the extent to which each domain is impaired by his or her symptoms on a 10-point visual analogic scale [85].

In addition, respondents were also invited to state via a free text comment whether they thought that the experience they had lived through had an effect on their children, or their relationship with them, and in what way.

##### Request for institutional and/or social support and awareness of resource persons

Assessment of social support was declarative. Respondents were asked whether they knew of “resource persons” in the institution likely to provide help with psychosocial risks and the level of social support received, on a scale of 1 to 5; whether in their personal entourage, there were persons on whom to rely in case of moral or emotional need, and/or of material need; more generally, whether they felt alone or not in life (with a gradation of 1 to 4 from ‘very much alone’ to ‘very well supported’).

#### Awareness of the specialised support facilities provided in the context of the survey, and care follow-up

Hospital managers and even physicians themselves may underestimate the effects of critical work-related incidents [11]. Screening for symptoms and subsequent follow-up should be systematically considered, particularly after a major traumatic event or repeated exposure to stress [58, 76, 86, 87].

In 2017, implementation of the *ECHOS de Nice* survey made it possible to outline hospital personnel care pathways. Since the web questionnaire might give rise to a revival of traumatic events in certain respondents, new support facilities were offered. At the end of the questionnaire, participants who « might feel the need for psychological assistance » were asked if they would « agree to have an interview with a specialist in psycho-traumatic stress ». In case of an affirmative reply, they were asked if they agreed to be contacted and then to provide their contact details. The Nice University Hospital Public Health department was responsible for managing these requests on an entirely confidential basis. The Nice University Hospital Human Resources department allocated time for a clinical psychologist specifically trained in psycho-trauma to contact respondents from the CHU and Lenval hospitals who had expressed this need and to offer an appointment for specialized management according to their needs assessment.

The survey aimed to determine the proportion of staff members who accessed psychological support and care follow-up, particularly those respondents whose mental health was affected and who required appropriate management [51, 88]. The medical profession is notoriously reluctant to consult, particularly regarding matters relating to mental health [89–92].

### Statistical analysis

Qualitative data were described using mean and standard deviation, and qualitative data were described using frequency and percentage. Comparisons between the different groups were made using the Student or ANOVA test for quantitative variables, and using the Chi2 test or Fisher’s exact test for qualitative variables. A *p*-value < 0.05 was considered significant.

A multivariate analysis was performed using logistic regression. Factors with a *p*-value < 0.20 in the univariate analysis were retained for model construction. Variables with a *p*-value < 0.05 were retained. 95% confidence intervals were provided. Statistical analyses were performed using the SAS Enterprise Guide 7.1 software.

### Free-text comments

The experience gained from the *IMPACTS* and *ESPA 13 novembre* studies illustrated the significant need of respondents to freely express themselves on various themes related to the attack and their substantial contribution in doing so. Based on this experience, the *ECHOS de Nice* questionnaire offered broader space for free comments which could be added for all the topics addressed: circumstances of exposure and memorable features, personal experience with consequences relating to several aspects of daily life (implying relationships with children), psychological and behavioural changes, circumstances of psychological support, level of satisfaction and suggestions for improving such support, acknowledgement of completed tasks by the institution. In particular, free comments pertaining to care follow-up may provide clues for improving the quality of services offered to professionals confronted with disasters or complex emergency situations [93].

## Discussion

To our knowledge, few studies focusing on the repercussions on hospital staff of a terror attack have been published to date. Luce et al. studied the consequences of the Omagh bomb attack in Ireland on hospital personnel in terms of risk of PTSD [74]. Publications have also described the effects experienced by hospital staff in the context of war, e.g. the Gaza war [53], or missile strikes targeting a hospital [81] or in the context of a critical incident [11]. The impact of the 2015 Paris attacks on medical and psychological support teams [9, 25] was reported in recent publications, and more specifically on health professionals, including hospital, emergency psychological and medical services staff [10, 26], who intervened in the 13 November 2015 attack. Our study concerns all hospital departments, regardless of staff category.

Due to the unpredictable character of the event, research focusing on the consequences of broad-scale traumatic events has two distinctive features: studies are observational by nature, and assessments are conducted after the occurrence of the event (*post-only designs*), which restricts the possibility of ruling out pre-existing symptoms or previous exposures, thus potentially compromising study findings and confounding symptom reports with exposure levels [94].

The second characteristic relates to the necessity of rapidly designing and implementing the survey to limit recall bias [77], which constitutes an organisational challenge for research teams [94]. Although we relied on the expertise acquired by Santé Publique France during the Paris attacks [9, 10, 51], our survey began 11 months after the Nice attack. There were several reasons for this delay: tailoring the questionnaire to the specific context of a hospital institution, with multiple professional categories, various professional circumstances of exposure, testing and validating the model on a sample of hospital staff. Moreover, the study was conducted in two institutions, Nice University Hospital and Fondation Lenval, which required the involvement of several management and administration departments, and thus the necessary time for its implementation. Time was also required to obtain approval from the Ethics Committee [95]. Lastly, a communication strategy was developed and deployed towards the staff prior to initiating the study, encouraging participation and specifying the neutral and objective character of the study.

Such a delay may have led to recall bias, and changes in symptoms over time. Conversely, it may have had positive consequences: PTSD identified 11 to 15 months after the attack revealed chronic [48, 96] or delayed PTSD [15], offering the possibility to compare symptom prevalence rates among staff in Nice with those observed among those in Paris [10, 25]. The delay is also potentially useful to reveal professionals’ registration for care follow-up.

Constraints related to delay and cost also contributed to the choice of a secured Web-based questionnaire that allowed rapid data collection [94]. Web-based health-related research has advantages in terms of feasibility [97] even though participation rates can vary [98]. An online questionnaire is known to allow rapid surveys following mass traumatic events and appears to reduce the risk of bias linked to social desirability [94], a bias that can be found in studies among hospital professionals questioned on their own health. However, a web-based questionnaire requires certain precautions. On the technical level, anonymity must be insured and technical difficulties anticipated and overcome [99]: for the *ECHOS* survey, anonymity was guaranteed thanks to a separate telephone number database, unrelated to identifiers, and independent from the data processing department; in case of technical difficulties accessing the questionnaire, a telephone number was available to quickly solve the problem. On the epidemiological level, due to potential selection bias of respondents, as the questionnaire was accessible online, individuals not part of the hospital staff were theoretically able to access it. To limit this possibility, it was decided not to relay the information via the press so that awareness would be restricted to hospital personnel; furthermore, an initial filter screened the respondent in terms of eligibility.

A self-selection bias may have introduced a further limitation to the survey since individuals are inclined to favour questionnaires that relate to their personal experience [100], such as those exposed to the consequences of a terror attack, while those not exposed may have felt unconcerned by the survey. Conversely, exposed and psychologically affected professionals may have been reluctant to take part in this type of research, which can lead to a lack of representativeness of the study population [94, 101]. Lastly, members of staff may have left the institution between the terror attack and June 2017. This could lead to a “healthy worker effect” selection type bias. Although we do not have any information on the number and profiles of employees who left hospital services since the date of the terror attack, it is likely that some of them have left because of their experience of the attack. This could lead us to underestimate our results.

To the extent that health-related activities usually confront hospital professionals with numerous causes for stress, predisposing them to psychological risks and burnout [80, 102–104], provision of care to victims of a terror attack is an extremely violent event that intensifies these health-related risks. Such an impact on hospital professionals may go beyond simple health repercussions to compromise quality of care, risk of work stoppage and turnover of affected teams [11, 105, 106], an issue which should be of particular concern for institution managers. However, institutions lack clear instructions in terms of procedures and interventions to be implemented in favour of employees that are victims of a trauma event [107]. This study aims to contribute recommendations so that in case of collective events of a magnitude comparable to the Nice terror attack the consequences of its impact on health professionals exposed to critical situations may be anticipated and allow appropriate decisions to be made in due time [87, 108].

## Data Availability

Data are currently undergoing analysis.

## Abbreviations

A&E: Accident and emergency
HAD: Hospital anxiety and depression scale
OTP: One-Time Password
PCL5: *PTSD* Checklist for DSM V
PTSD: Post-traumatic stress disorder
